# Dynamic tethering of M protein drives pathological inflammation during group A *Streptococcus* infections

**DOI:** 10.1371/journal.ppat.1014434

**Published:** 2026-07-15

**Authors:** Ananya Dash, Stephanie Guerra, Doris L. LaRock, Richa S. Varughese, Paola Vidal, Alison Swaims-Kohlmeier, Christopher N. LaRock

**Affiliations:** 1 Department of Microbiology and Immunology, Atlanta, Georgia, United States of America; 2 Department of Gynecology & Obstetrics, Atlanta, Georgia, United States of America; 3 Department of Medicine, Division of Infectious Diseases, Emory School of Medicine, Atlanta, Georgia, United States of America; Murdoch Children's Research Institute, AUSTRALIA

## Abstract

The M protein of Group A *Streptococcus* (GAS) is an essential virulence factor that promotes both superficial and invasive infections, as well as the development of immune sequela, including acute rheumatic fever and rheumatic heart disease. M protein is a prototypical sortase-anchored surface protein, yet has also been observed free of the GAS surface during human infection. This suggests a mechanism for its release, and this change in localization could influence innate immune detection, effector functions, and antigenic potential. Here, we show the protease SpeB cleaves M protein from the microbial surface, releasing a nearly full-length fragment that is highly resistant to any further degradation. Leveraging this insight to engineer strains where M protein either remains surface-locked or is constitutively secreted, we examine their separate contributions to disease. Only the secreted form of M protein drives inflammation in a mouse model of invasive infection, contributes to neutrophil activation and influx, a hallmark of these pyogenic infections. Furthermore, the release of M protein contributes to increased bacterial survival, suggesting the importance of maintenance of this mechanism within the species. This study thus highlights the potential relevance for proteolytic regulation of surface proteins in bacterial pathogenesis.

## Author summary

While most pathogens subvert immune responses, group A *Streptococcus* is unique in its ability to amplify immune responses to its benefit. Here we study a mechanism for release of an abundant surface protein and in turn, its role in inflammation observed during infection. We show that surface M protein is released from the bacterial surface by the GAS protease SpeB. We identify the target sites of SpeB within M protein and use that information to engineer M protein variants of GAS that are uncleavable or that cannot be anchored to GAS. M protein, free from GAS surface, serves as a bait for neutrophils, enhances GAS proliferation, and induces worse pathology. On the other hand, when M protein is anchored to GAS surface, the outcomes are reversed with a reduction in recruitment of neutrophils and improved bacterial clearance. Our results delineate how GAS can vary the localization of a surface anchored protein to modulate innate immune responses.

## Introduction

The obligate human pathogen Group A *Streptococcus* (GAS, *Streptococcus pyogenes*) is a remarkably versatile pathogen adept at subverting host defenses. Superficial GAS infections, such as strep throat or impetigo, can progress to invasive infections and immune-driven sequelae like rheumatic heart disease. While a healthy immune system can resolve milder GAS infections, severe invasive ones such as necrotizing fasciitis require aggressive medical management [1]. During these infections, inappropriate immune cell activation and excessive inflammation are responsible for pathological complications, including the development of toxic shock syndrome and sepsis [2,3]. Antibiotic treatment can fail, in part a consequence of the coagulopathy, edema, and tissue and vasculature damage limiting drug penetration [4]. Hence, there is a need to improve our understanding of how GAS triggers improper immune responses to prevent life-threatening outcomes.

M protein is the most abundant protein on the GAS surface, existing as an α-helical coil-coil dimer anchored to cell wall peptidoglycan through an LPxTG motif and extending as dense, hair-like cell surface appendage [5–8]. The hypervariable N-terminal region of M protein protrudes furthest from the cell surface and comes in greater than 275 variants, forming the basis of *emm* typing of GAS [9]. M proteins bind number of host proteins including fibrinogen, plasminogen, C4b-binding protein, immunoglobulins, cathelicidin (LL-37), and histones within neutrophil extracellular traps, and consequently, provide antimicrobial resistance, mask the GAS surface, and promote cell and tissue adhesion to host [7,10–17]. Additionally, M protein is proposed to be a molecular mimic of the structurally-similar human proteins myosin, tropomyosin, and keratin, and through these proteins as well as binding substrates such as collagen, can lead to generation of cross-reactive antibodies responsible for autoimmune rheumatic diseases [18–20]. M protein activity can vary between alleles, by the presence of distinct host factors at the infection site, and, possibly, by localization, since M protein is not always anchored to GAS surface and both host and microbial proteases have been implicated in its release [21–23].

The bacterial protease SpeB was discovered for its interference with serotyping, which is classically based on antibodies against the variable N-terminal region of M protein [21]. Further consistent with cleavage, SpeB can also interfere with M protein-dependent binding of fibrinogen and adherence to epithelial cells [22,24]. After release from cells, M protein may also have activity. It has been observed non-colocalizing with GAS within samples from human infection, suggesting some measure of stability and abundance, and this may be driven in part in by neutrophils [23]. Subsequent studies show that recombinant M protein itself is sufficient to be highly proinflammatory. Specific mechanisms for this proinflammatory activity include inducing pathological neutrophil degranulation [25,26] and secretion of heparin-binding protein, which contributes to vascular leakage during severe infection [23,25,27]. Both neutrophils and monocytes release proinflammatory cytokines on exposure to M protein [25]. In monocytes, this release is dependent on M protein interaction with TLR2 [25]. Macrophages activate the NLRP3 inflammasome in response to M protein internalization, and die by pyroptosis, secreting interleukin (IL)-1β and IL-18 in the process [28]. Platelets are activated by M protein, which aggregate and form a thrombus, also as seen during infection [12,29,30]. M protein also induces a Th1 type response from T cells, with elevated IL-1β and granulocyte-macrophage colony-stimulating factor (GM-CSF), which have established pivotal roles in autoimmunity [31–35].

Despite these myriad activities, the contribution of SpeB-mediated release of M protein from the bacterial surface to pathogenesis, and if it even occurs during infection, was unknown. This study shows that M1 protein maintains a conserved cleavage site targeted by SpeB and is released from the cell surface in a nearly full-length form that is resistant to further degradation. This is consistent with the stability that would be required for it to retain biological activities after its release. GAS with M1 protein constitutively locked on its surface is attenuated similarly to GAS lacking M protein entirely, and induces less inflammation. These data support a mechanism where dynamic regulation of the cell surface by a bacterial protease is important for pathogenesis. Maintaining protein on the surface can thus promote cell adhesion and masking of the cell by captured host proteins, while its cleavage allows greater interaction with immune cells and molecules away from the bacterial surface. The ability to get differing effects from a single protein through a post-translational modification can thereby allow GAS to adapt to the infection environment.

## Results

### M1 protein is released from the cell surface

**Fig 1:**
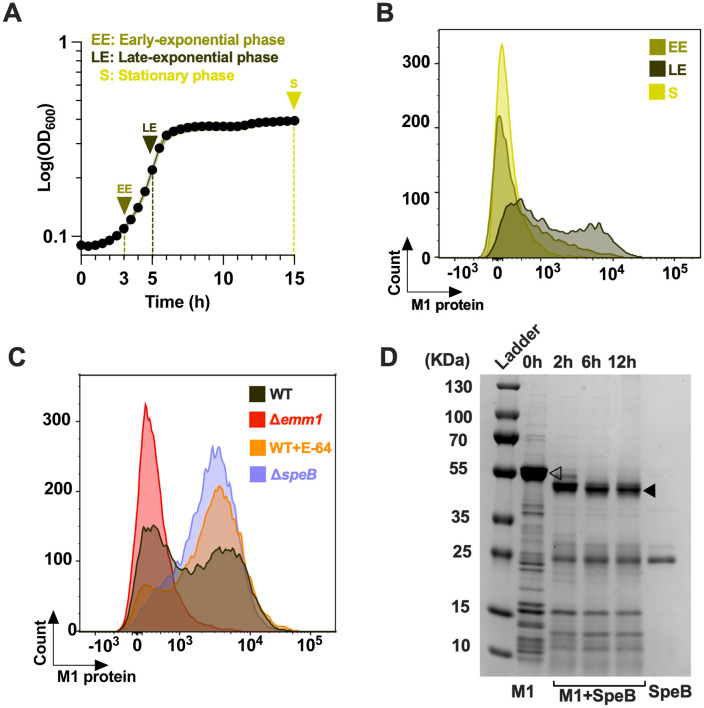
SpeB releases M1 protein from the GAS surface. **(A)** Growth curve of 5448 GAS wildtype (WT) indicating time points of sample collection for flow cytometry in **B. (B)** Surface M1 protein measured in WT grown to early-exponential phase (EE), late-exponential phase (LE), and stationary phase (S) by flow cytometry. **(C)** Surface M1 protein measured in WT, Δ*emm1*, and Δ*speB* grown to late-exponential phase by flow cytometry. Addition of SpeB inhibitor, E-64 at 200 μM to WT prevents M1 protein release from GAS surface. M1 protein release is SpeB-dependent, as shown in Δ*speB*. **(D)** SpeB releases a distinct stable product (solid triangle) from recombinant M1 protein (open triangle), separated by SDS-PAGE and visualized by staining. Data are representative of three independent experiments.

Using flow cytometry, we measured surface expression of M protein during different growth phases **(Fig 1A)** of GAS strain 5448, representative of the global M1T1 clone that is a major driver of severe disease [36,37]. Surface M1 protein levels started to rise at an early-exponential phase, yielded a bimodal distribution by late-exponential phase, and declined at stationary phase in wildtype (WT) GAS **(Fig 1B)**. This pattern led us to examine the role of SpeB, since *speB* expression *in vitro* is strictly limited to stationary phase and thus commonly absent in most studies as bacteria are typically sub-cultured to early- or mid-exponential phase before use [38–40]. Late-exponential GAS grown in media containing E-64, an inhibitor of SpeB [39], eliminated the bimodal distribution of surface M1 protein and increased the retention of M1 protein on the surface in WT GAS **(Fig 1C)**. Next, we confirmed this genetically by quantifying surface M1 protein in a GAS mutant unable to express SpeB. *ΔspeB* GAS retained M1 protein on the GAS surface, showing that SpeB is necessary and sufficient to control the release of M1 protein during *in vitro* growth **(Fig 1C)**. To confirm direct cleavage, SpeB was incubated with purified M1 protein and examined by SDS-PAGE, yielding a truncated a 47 kDa form **(Fig 1D)**, consistent with observations that M1 protein is stable, has a long half-life *in vivo*, and may be able to bind proteins after its release [23,26,41]. Taken together, these results point to a dynamic presence of M1 protein on the GAS surface and show that SpeB expression dictates its retention or release *in vitro*.

### SpeB cleaves M1 protein proximal to the sortase anchor

**Fig 2:**
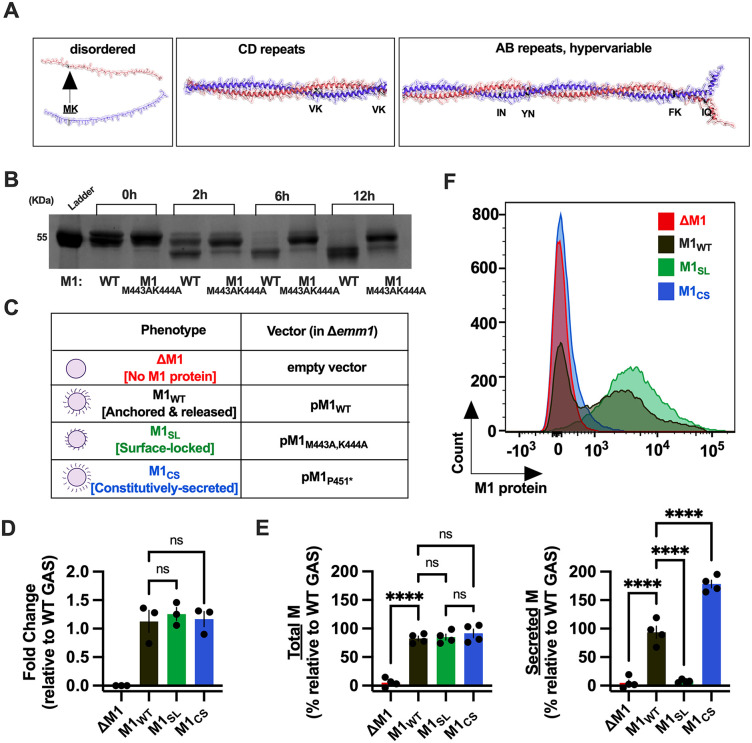
SpeB targets M1 protein at the C-terminal end proximal to the GAS surface. **(A)** Cleavage site prediction of SpeB in M1 protein; MK_444_ (underlined) is a site of SpeB cleavage, VK, IN, YN, FK, and IQ are potential sites of cleavage not observed. **(B)** Alanine substitutions (M1_M443AK444A_) render the M1 protein uncleavable by SpeB compared to wildtype M1 protein (WT). **(C)** 5448 GAS Δ*emm1* transformed with pDCErm, pDCErm-emm1, pDCErm-emm1_M443AK444A_, pDCErm-emm1_P451*_ respectively and referred to as ΔM1, M1 _WT_, M1_SL_ (surface-locked) and M1_CS_ (constitutively secreted). **(D)** qRT-PCR measurements of *emm* gene from ΔM1, M1 _WT_, M1_SL_, and M1_CS_. **(E)** ELISA measurements of culture (total) and supernatant (secreted) M protein from ΔM1, M1 _WT_, M1_SL_, and M1_CS_. **(F)** Surface M1 protein detection in ΔM1, M1_WT_, M1_SL_, and M1_CS_, by flow cytometry. Data are representative of three independent experiments. Statistical analysis was done by one-way ANOVA with Tukey’s multiple comparisons test. ****p ≤ 0.0001, ns, no significance.

M1 protein extends from the surface of GAS cell wall primarily as a tight α-helical coil-coil [16], a structure that is highly stable and generally resistant to proteolysis [42]. Alphafold modeling recapitulates this structure and also shows the ~ 54 amino acids most proximal to the surface are disordered **(Fig 2A)**, consistent with prior biochemical data [16]. Prediction of possible SpeB cleavage sites based on known substrates, as previously [43], suggested SpeB cleavage occurs after K444 **(Fig 1D)**, matching the observed product. Additional amino acid pairs matching cleavage sites observed in gasdermins and other targets are present **(Fig 2A)**. However, these were present in coil-coil regions and in locations where amino acid side chains were not predicted to be accessible for cleavage, explaining why no cleavage at these sites was observed by SDS-PAGE in the formation of products with corresponding molecular weights **(Fig 1D)**. Based on prior studies, alanine substitution of the two amino acids N-terminal to a cleavage (P1 and P2 sites) prevents SpeB proteolysis of a substrate [39]. Substitution of residues 443–444 (M1_M443A,K444A_), abolished SpeB cleavage, generating a form of M1 protein stabilized against cleavage by SpeB (**Fig 2B**).

Accordingly, we expected GAS expressing M1_M443A,K444A_ to retain greater M protein on the bacterial surface. For comparison, the construct M1_P451*_ was also generated, where truncation of the C-terminus to eliminate the LPXTG motif and abolish sortase A processing was expected to result in protein that is secreted and never anchored [5]. *Δemm1* GAS was complemented with plasmid to express the non-mutant M1 protein (M1_WT_), the noncleavable M1_M443A,K444A_ mutant (M1_SL_; surface-locked), or M1_P451*_ (M1_CS_; constitutively secreted) (diagramed in **Fig 2C**, see **Table 1** for primers). Gene expression analysis of *emm1* gene by qRT-PCR from engineered plasmids showed no difference when M1_SL_ and M1_CS_ were compared to M1_WT_ (**Fig 2D**) or difference in growth (**S1 Fig**). Measurement M protein by enzyme-linked immunosorbent assay (ELISA) shows equivalent levels of total levels among M1_SL_, M1_CS_ and M1_WT_, but greater release into the supernatant by M1_CS_ relative to M1_WT_, and little by the M1_SL_ or ΔM1 (**Fig 2E**). By flow cytometry, M1_SL_ showed surface retention of M1 protein and M1_CS_ showed no M1 protein, comparable to the non-complemented *Δemm1* mutant (ΔM1) (**Fig 2F**). Thus, each of these genetic constructs recapitulates a population of the bimodal distribution of M1 protein observed for M1_WT_ (**Fig 2F**) and WT GAS **(Fig 1C)** and can be used to control M protein anchoring independent of SpeB for further examination on how the protein’s localization impacts its activities as a virulence factor.

**Table 1:** Primers for cloning.

Primer	Sequence (5’-- > 3’)
pET-SUMO Emm1 C	ACCACCAATCTGTTCTCTGTGAG
pET-SUMO Emm1 D	GGCCGCACTCGAGCAC
pET-SUMO Emm1 A	CTCACAGAGAACAGATTGGTGGTATGG CTGTTAAGGCTAACGG
pET-SUMO Emm1 B	TGCTCGAGTGCGGCCTTAGTTTTCTTCTTTGCGTTTTACAACTG
pDCErm-Emm1-M444A K444A A	CCAGCGGCGGAAACTAAGAGACAGTTACCATCAA
pDCErm-Emm1-M444A K444A B	TTCCGCCGCTGGTGCTTTGTTTTGGTTAGG
pDCErm-Emm1-P451* A	TTATGATCAACAGGTGAAACAGC
pDCErm-Emm1-P451* B	TGATCATAACTGTCTCTTAGTTTCCTT

### Released M1 protein is cytotoxic and inflammatory to macrophages

**Fig 3:**
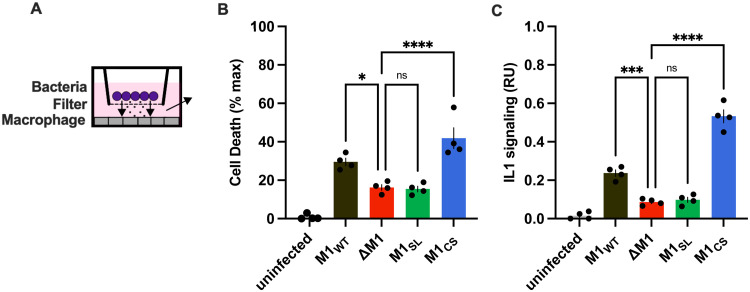
GAS induces cell death upon M1 protein release. **(A)** Schematic of GAS migration towards THP-1 cells. **(B)** Cell death was measured by lactate dehydrogenase release **(C)** Total and active IL-1β production by GAS-infected THP-1 cells 4 hours after infection via IL-1R reporter assay. RU, relative unit. Data are representative of three independent experiments and are expressed as the mean±SEM of four technical replicates. Statistical analysis was done by one-way ANOVA with Dunnett’s multiple comparisons test. *p ≤ 0.05, **p ≤ 0.01, ***p ≤ 0.001, ****p < 0.0001.

Recombinant M protein has been shown to be sufficient to induce pyroptosis in macrophages, resulting in cell death and the activation of IL-1 signalling [28]. However, this has not been examined with bacteria, where the quantity M protein and the presence of additional bacterial factors could influence its immune detection. To examine whether release of M1 protein from the GAS surface, or its retention, impacts pyroptosis, each clone was incubated with macrophages with a 0.4 µm membrane separating the two, preventing direct cell-cell contacts (**Fig 3A**). After 4 hours, macrophage lysis (**Fig 3B**) and IL-1 signaling (**Fig 3C**) were measured. All strains induced some M protein-independent lysis (**Fig 3B**), consistent with contributions from SLO, SLS, SpeB, and other toxins that can also induce pyroptosis. However, M1_SL_ induced no more than a ΔM1 mutant, and M1_CS_ significantly more (**Fig 3B**). A similar trend was observed for the generation of bioactive IL-1 (**Fig 3C**). Therefore, M1_SL_ has a more limited proinflammatory capacity. In contrast, released M1 protein has the capacity to induce inflammation in cells that are not even in direct contact with GAS.

### Released M1 protein drives innate immune cell recruitment

**Fig 4:**
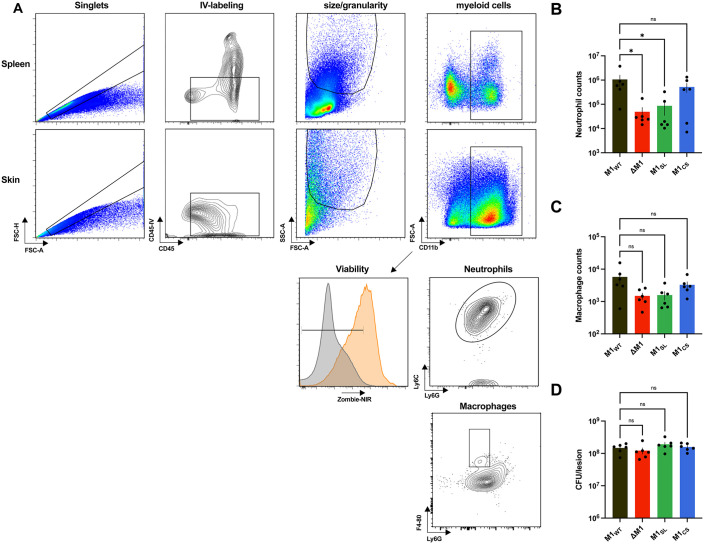
M1 protein release shapes recruitment of innate cells during infection. Wild-type (C57Bl/6) mice were inoculated intradermally with M1_WT_, ΔM1, M1_SL_, and M1_CS_ for 24 hours. **(A)** Flow cytometry cell gating strategy for measuring innate immune cells including neutrophils and macrophages in skin, compared with spleen. **(B)** & **(C)** Counts of neutrophils and macrophages per gram of tissue lesion. **(D)** Colony-forming units (CFUs) of GAS at the site of infection were measured by plating. Data are expressed as the mean±SEM (n = 6). Statistical analysis was done by one-way ANOVA with Dunnett’s multiple comparisons test for B and C and Tukey’s multiple comparisons test for **D.** *p ≤ 0.05, **p ≤ 0.01, ns, no significance.

To examine how M1 protein release from the surface impacts bacterial fitness, we utilized an established model of intradermal infection to measure GAS growth in necrotic skin ulcers [2,39]. To better understand how early sensing of GAS impacts the innate immune response, we performed intravital vascular (IV) staining prior to necropsy to label and distinguish circulating leukocytes from those in the infected tissue and analyzed these populations using flow cytometry 24 hours post-infection (**Fig 4A, see Table 2 for antibodies**). Significantly fewer neutrophils were recruited during infections by M1_SL_ and ΔM1 strains (**Fig 4B**). No differences in macrophages were observed during infections among groups (**Fig 4C**). Since there were not significant differences in bacterial number at this time point (**Fig 4D**) or M protein expression (**S2 Fig**), we conclude that it is not the presence of M protein, but whether it is secreted, that is a critical driver of the influx of neutrophils during infection.

**Table 2:** Antibodies used for flow cytometry.

Marker	Clone	Primary Detector	Company & Catalog
PE-CF594 IV-CD45	30-F11	YG3	BD-H #562420
Spark Blue 574 CD45	30-F11	B4	BL #103184
Spark UV 387 Ly6G	1A8	UV1	BL #127678
BV650 F4/80	BM8	V11	BL #123149
PerCP/Cyanine5.5 CD11b	M1/70	B9	BL #101228
PE/Cyanine7 CD63	NVG-2	YG9	I #25-0631-82
AlexaFluor 700 Ly6C	HK1.4	R4	BL #128024

### Impacts of M protein on neutrophil activation

**Fig 5:**
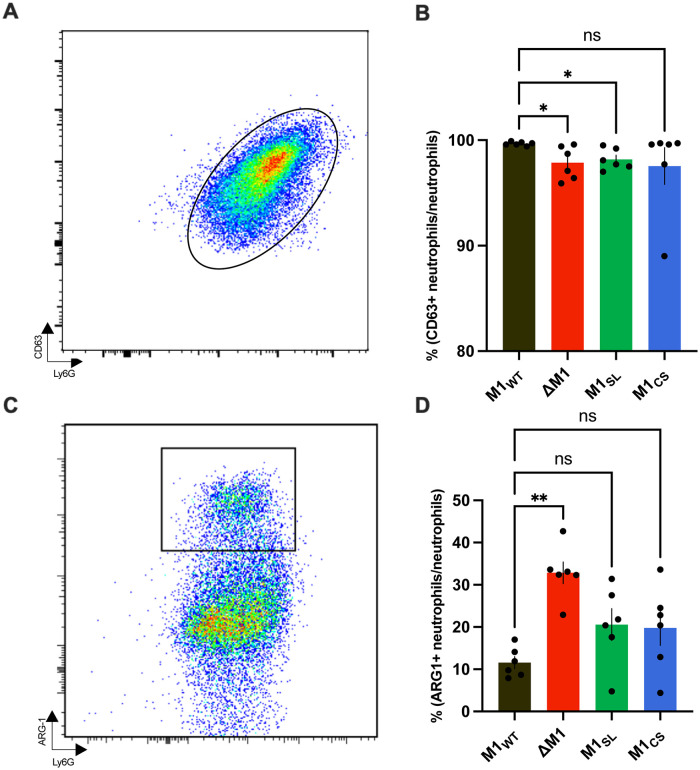
M1 protein release reprograms neutrophils. Wild-type (C57Bl/6) mice were inoculated intradermally with M1_WT_, ΔM1, M1_SL_, and M1_CS_ for 24 hours. **(A)** Neutrophils gated for CD63. **(B)** Percent frequency of neutrophils expressing CD63 marker. **(C)** Neutrophils gated for ARG1. **(D)** Percent frequency of neutrophils expressing ARG1 marker. Data are expressed as the mean±SEM (n = 6). Statistical analysis was done by one-way ANOVA with Dunn’s multiple comparisons test for A and **B.** *p ≤ 0.05, **p ≤ 0.01, ns, no significance.

To better understand not just the quantitative differences in recruitment of innate cells, but if there were qualitative changes in cell activity, we examined tissue neutrophils for additional markers of activation and differentiation. The frequency of neutrophils displaying the CD63 **(Fig 5A),** a marker of degranulation, was reduced in mice infected with M1_SL_ and ΔM1 (**Figs 5B**, **S3****A)**. On the other hand, the frequency of neutrophils displaying this marker stayed comparable in mice infected with M1_CS_ and M1_WT_ (**Figs 5B**, **S3****A)**. While we observe a role for released M1 protein to contribute to the expression of CD63 in neutrophils, the overall frequency of CD63 + neutrophils was comparable across our groups.

Next, we measured the frequency of neutrophils expressing Arginase-1 (ARG1) (**Fig 5C**)**,** which catalyzes the conversion of arginine to ornithine and can thereby suppress responses of T cells and immune cells [44–46]. An overall increase in frequency of ARG1^+^ neutrophils was observed in mice infected with ΔM1 as compared to M1_WT_ (**Figs 5D**, **S3****B)**. No differences in frequency of ARG1 + neutrophils were observed when mice infected with M1_SL_ or M1_CS_ were compared to mice infected with M1_WT_ (**Figs 5D**, **S3****B)**. These results show that, in the absence of M1 protein, GAS creates an immunosuppressive state in the tissue and that this is independent of M1 protein localization.

### M1 protein release is pathogenic

**Fig 6:**
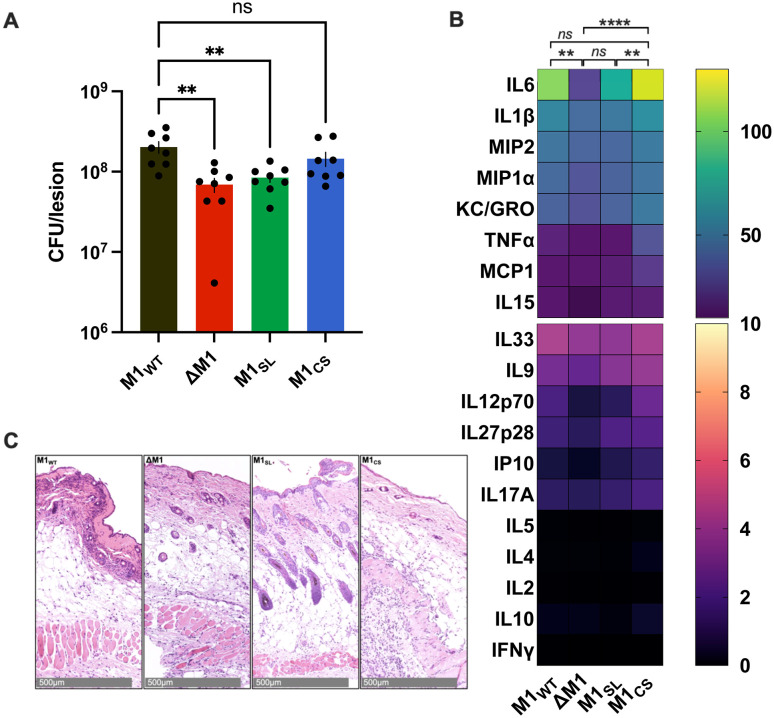
M protein release worsens skin pathology. Wild-type (C57Bl/6) mice were inoculated intradermally with M1_WT_, ΔM1, M1_SL_, and M1_CS_ for 72 hours. **(A)** Colony-forming units (CFUs) of GAS at the site of infection were measured by plating. **(B)** Heatmap of pro-inflammatory cytokines from cell-free lysates from site of infection were measured postinfection by V-PLEX proinflammatory cytokine panel 1 (MSD). **(C)** Skin was examined by Hematoxylin and Eosin stain. Data are expressed as the mean±SEM (n = 8). Statistical analysis was done by one-way ANOVA with Tukey’s multiple comparisons test for A and Dunnett’s multiple comparisons test for **B.** *p ≤ 0.05, **p ≤ 0.01, ***p ≤ 0.001, ****p < 0.0001, ns, no significance.

**Fig 7:**
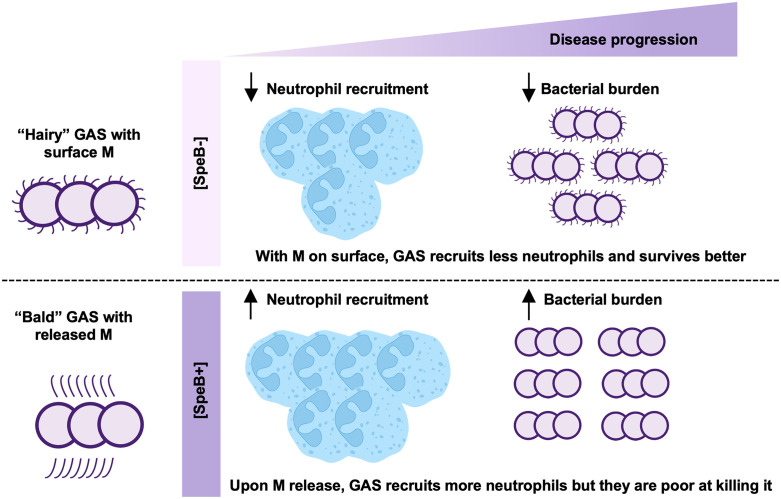
Model of M1 protein release. GAS protease SpeB releases M1 protein from the bacterial surface. During severe infection, more neutrophils arrive at the site of infection as a result of M1 release and mount a pro-inflammatory response. However, they are insufficient in reducing the overall bacterial burden. When M1 is locked on GAS surface, fewer neutrophils respond to the infection, but GAS is better controlled. (Neutrophil image attribution: Helicase11, Bioicons, CC-By 4.0).

While no significant difference in GAS growth was observed within 24 hours, these differences in immune activation had the potential to eventually impact bacterial replication and pathology if the infection was allowed to continue. Consistent with the attenuation observed in previous studies [14], after 72 hours there was significantly less survival of ΔM1 GAS. Interestingly, the bacterial burden was reduced in mice infected with M1_SL_, but not M1_CS_, which was recovered at numbers comparable to M1_WT_ **(Fig 6A)**. Thus, having the ability to release M protein into the tissue from the GAS surface modestly contributed to GAS survival. Concurrent with this, infection by the M1_CS_ strain also induced a significantly greater proinflammatory response than ΔM1 or M1_SL_, with elevated IL-1β, IL-6, TNF-α, IL-17A, MIP-1α, MIP2, MCP-1, IP-10, KC/GRO, IFN- γ, IL-2, IL-4, IL-5, IL-9, IL-10, IL-12p70, IL-15, and IL27p28 **(Figs 6B,** **S4**) and tissue damage **(Fig 6C)**. In contrast, the immune response was diminished in mice infected with M1_SL_ relative to M1_WT_ (**Fig 6B**). Together, these data demonstrate that the tethering or secretion of M1 protein impacts bacterial survival and the immune response, with the secreted form having both proinflammatory and provirulence consequences for GAS (**Fig 7**).

## Discussion

After a century of studies on M protein, the immune subversion mechanisms of M protein continue to expand and inform our understanding of streptococcal diseases [7]. M protein has been assumed to primarily be surface-anchored, and that it is from this vantage that it would promote adherence and interfere with complement, antimicrobial peptides, and its other targets [11,14,24,29,30,41,47,48]. Proteomics with recombinant M protein shows binding can be maintained even when the protein is not on the cell surface [8,49], and experimental evidence gives potential biological roles specific to released protein, such as forming networks with fibrinogen, stimulating neutrophil degranulation, and activating the inflammasome [15,23,25–28]. Expanding on prior observations, we demonstrate that the release of an intact form of M protein from the cell surface is regulated by another bacterial virulence factor, the protease SpeB. M protein is typically one of the most abundant surface proteins, and SpeB is one of the most highly expressed proteins in human and animal models of GAS infection [50–52]. Each individually contributes to virulence in multiple ways, but using engineered strains, we can isolate the contribution of this singular cleavage event to pathogenesis. Across a variety of conditions, we observe diversity in GAS populations, with subpopulations either harboring M protein on the surface or shedding M protein. Each of these has differing potential to shape immune responses, impacting their individual fitness and that of the population.

Neutrophils are the major responders to infection [53,54], and we show that released M protein is the primary driver of neutrophil recruitment and activation (**Fig 4B**). These neutrophils can induce expression of SpeB, potentially amplifying these responses [55]. M protein-fibrinogen complexes via binding to β2-integrins [23] or forming aggregates with platelets may also make neutrophils hyper responsive [56]. Furthermore, Snäll et al., has showed recombinant M protein increases secretion of neutrophil-derived molecules associated with acute inflammatory conditions [27]. During infection, we also observe bacterially-expressed M protein, upon its release, also recruits large number of neutrophils (**Fig 4B**) that degranulate (**Fig 5B**). These have the potential to further increase release of M protein from the surface [23]. Yet, bacterial growth remains similar between mice infected with M1_WT_ and M1_CS_, suggesting neutrophils become ineffective in bacterial clearance by actively favoring to respond to only M protein released from GAS (**Fig 6A**). This adds to the existing understanding of how M protein, despite contributing to neutrophil activation, is also important for GAS in resisting neutrophil killing [14,57].

We made another interesting observation about neutrophils when we compared the frequency of neutrophils expressing ARG1 during our infection. Lack of M1 protein in GAS led to an increase in ARG1 + neutrophils (**Fig 5D**) and could be indicative of a shift in neutrophils towards a more anti-inflammatory and immunoregulatory state. ARG1 can inhibit T-cell activation [58], but also, GAS is auxotrophic for arginine and arginine catabolism is one of the most induced in GAS during an epicutaneous infection of mice [50]. Arginine restriction also promotes antibiotic tolerance in GAS [59,60]. Hence, when neutrophils increase ARG1 production and deplete arginine from the surrounding tissue, it could be detrimental for GAS and our data (**Fig 6A**) shows that bacterial burden in mice infected with ΔM1 is least when compared to other groups. M1 protein could potentially modulate ARG1 in neutrophils to scavenge arginine for its dissemination. The mechanism could be similar to the need of GAS to sense and modulate asparagine, an amino acid that GAS relies to uptake from host [61,62]. But future studies will inform if M protein can act as a sole factor to impact bacterial growth and to what extent it can aid in resolution of infection.

Proteolysis is a post-translational regulatory mechanism that is not inherently destructive. For example, our recent studies show SpeB cleavage of some host proteins, such as proinflammatory cytokines and gasdermin-family cell death effectors, instead activates their functions, and that they are resistant to further degradation [43,63–65]. As a promiscuous protease, SpeB has the potential to also cleave bacterial proteins [39,63,64]. However, especially with large quantities of protease or extended incubations, cleavages are not always physiologically relevant nor necessarily destructive; superantigens, for example, are quite resistant to degradation by SpeB relative to their orthologs from *Staphylococcus aureus*, which does not encode a SpeB-like protease [43]. The amount of SpeB produced can thus impact substrate processing, and can vary throughout the host with antimicrobial peptide, pH, reducing environment, and other host and bacterial factors all potentially impacting SpeB expression and activity [66]. Thus, the timing of M1 protein cleavage is notable not just that it happens quickly, and in relevant conditions, but also that the protein remains stable over time, showing little degradation even after 12 hours of incubation (**Fig 1D**).

When GAS releases M protein and can benefit from this release may be context-dependent. First, not only is the cleavage site identified in our study not conserved across all *emm* types (**S5 Fig**), but each allele has the potential for cleavage at locations not observed for M1. This would make the balance between surface-bound and released M protein strain-specific. This balance is likely also infection-specific: *emm* mutants tend to be more attenuated in human whole blood and neutrophil killing assays than mouse skin infection models, suggesting additional selective pressures [14,15,67,68]. However, this whole blood killing attenuation is not seen for *emm* mutant GAS when in pooled transposon screen, suggesting some level of population-level complementation [69]. Consistent with this, mutants in *covS/csrS* or *ropB* can arise during invasive infections that prevent SpeB expression [40]. SpeB expression phenotype is maintained in the species [70], suggesting these suffer from an overall attenuation, but subpopulations retaining greater surface M protein could alter the infection course. A recent preprint describes a different sort of mutation impacting M protein anchoring: a clone that arose during experimental infection with an *emm* nonsense mutation [68]. This resulted in constitutive M protein release without the need for SpeB and, as in our study, was associated with virulence. However, as with *covS/csrS* or *ropB*, there is no evidence of fixation of mutants like this in the population, suggesting deficiencies in transmission or other essential features of natural infection. Thus, even if there are temporal or spatial virulence benefits to M protein release, doing so in a regulated manner may be most advantageous.

## Materials and methods

### Ethics statement

This study was conducted according to the principles expressed in the Declaration of Helsinki. Animal experiments were approved by the Institutional Animal Care and Use Committees of Emory University.

### Bacterial strains

GAS strain 5448, representative of the pandemic M1T1 clone, and its isogenic *Δemm1, ΔspeB*, and pDCErm-Emm1 complement have been previously described [14,39,71–73]. All bacteria were statically grown at 37℃ with 5% CO_2_ in Todd Hewitt broth (Difco) supplemented with 1% yeast (THY), supplemented with erythromycin (2 µg/ml) when needed for plasmid maintenance and E-64 (Sigma Aldrich) at 200 µm for inhibition of SpeB. Fresh cultures were grown in THY to stationary phase and early- or late-exponential phase using the overnight culture as the starter at 1/20^th^ dilution.

### Growth curve

GAS 5448 grown in THY to mid-exponential phase were used to inoculate fresh THY in a 96-well black, clear-bottom plate (Costar). Cultures were grown for 15 hours at 37°C and 5% CO_2_ with measurements of OD_600_ every 30 minutes using a BioTek Synergy H1 plate reader.

### Plasmids

pET-SUMO, a vector for expressing recombinant proteins with cleavable His-SUMO tag on the N-terminus, is previously described [64]. The coding sequence encoding the M1 protein without the signal peptide (1–38) was cloned from GAS 5448 into pET-SUMO using Polymerase Incomplete Primer Extension (PIPE) cloning technique into TOP10 and transformed into DH5α, creating pET-SUMO-Emm1 [74]. Point mutants were created by site-directed mutagenesis through PIPE cloning of this vector, creating pET-SUMO-Emm1_M443A,K444A_. pDCErm-Emm1_M443A,K444A_ and pDCErm-Emm1_P451*_ were similarly created in pDCErm-Emm1, which is previously described [57]. pDCErm, pDCErm-Emm1, pDCErm-Emm1_M443A,K444A_ and pDCErm-Emm1_P451*_ were transformed into 5448*Δemm1* and were designated as ΔM1, M1_WT_, M1_SL_, and M1_CS_ respectively*.* All plasmids were verified by sequencing. The list of primers used are listed here.

### Protein purification and characterization

The structure of mature M1 protein (42–451 of W0T3T8, lacking secretion signal and anchoring domain) was modeled as a dimer in AlphaFold Server v3 and visualized in ChimeraX v1.9. Sites of potential SpeB cleavage were predicted as previously described based on known targets [43] using ScanProsite (Expasy) with a search for the motif [IVFYM]-[ADEGKSTN] and the included condition for a net negative charge sidechain charge in the P1’-P5’ region.

Expression of recombinant M1 protein from pET-SUMO-Emm1 and pET-SUMO-Emm1_M443A,K444A_ plasmids were done in *E. coli* BL21 (DE3) induced with 0.1 mM isopropyl-β-d-thiogalactopyranoside (IPTG, Sigma) at 16°C overnight in 1 L Luria Broth. Bacterial cell pellets were suspended in 10 mL of phosphate-buffered saline (PBS, pH 7.4), then lysed by sonication at 11% amplitude (Sonic Dimembrator Model 500, Fischer Scientific) for four minutes for 30 seconds at 30 seconds intervals and centrifuged at 7500 x g for 10 minutes at 4°C. Filtered, clarified culture was passed through Talon gravity columns loaded with HisPur️ Cobalt Resin (Thermo Scientific), washed with PBS, eluted in PBS supplemented with 300 mM imidazole (Sigma), and dialyzed using SnakeSkin Dialysis tubing (Thermo Scientific). The His-SUMO tag was removed by digestion with ULP1 at 4°C. The His-SUMO fragment was removed using HisPur Cobalt Resin, and M1 was obtained in the flow-through fraction. Samples were evaluated for purity by SDS-PAGE. SpeB was purified as previously [39] and confirmed to be > 99% pure by SDS-PAGE. The specific activity of SpeB was measured by incubation with the specific FRET peptide Mca-IFFDTWDNE-Lys-Dnp (CPC Scientific) in PBS with 1 mM dithiothreitol and measuring the change in fluorescence, as previously [39].

### M1 and M1 M443A K444A protein cleavage

Purified recombinant M1 (0.7 mg/ml) and M1_M443A,K444A_ (0.7 mg/ml) was incubated with SpeB (0.1mg/ml) in assay buffer (PBS, 2 mM dithiothreitol) for the indicated times at 37°C, as previously [39]. Reactions were stopped by the addition of Laemmli buffer (Bio-Rad) and 10% 2-mercaptoethanol (Bio-Rad), then boiled at 98 ℃ for 5 minutes. Samples were analyzed by SDS-PAGE on Tris-Glycine gels (Invitrogen) and visualized with AquaStain (Bulldog Bio).

### Cell surface expression of M1 protein using flow cytometry

For cell surface detection of M1 protein, GAS strains were grown for 5 hr to late-exponential phase (indicated in **Fig 1A**), centrifuged at maximum speed for 5 minutes, and resuspended in PBS with 1 mM ethylenediaminetetraacetic acid (EDTA) buffer. The samples were filtered with 70 µm cell strainer (Avantor), washed in PBS-EDTA, and fixed with 4% paraformaldehyde (PFA, Electron Microscopy Sciences) for 30 minutes. M1 protein on the GAS surface was detected using anti-M1 IgG from pooled serum isolated from rabbits immunized against the J8 epitope of M1 protein [75,76]. Samples were washed twice before adding anti-M1 IgG in 1:1000 dilution to the samples and incubated at 4°C overnight. Samples were washed and incubated with Alexa Fluor 647 Goat Anti-Rabbit IgG (H + L) cross-adsorbed secondary antibody (Life Technologies, Catalog A21244) at 1 µg/ml for 30 minutes. Samples were washed and filtered with 40 µm cell strainer (Avantor) before analysis using Fortessa X20 (BD Biosciences) flow cytometer and at least 10000 events analyzed for each condition. Bacterial samples were processed for an unstained control and a *Streptococcus* group A carbohydrate antibody (Fitzgerald) labeled with Alexa Fluor 647 Goat Anti-Rabbit IgG (H + L) as an Alexa Fluor 647 control.

### Measurement of M protein by ELISA

A microtiter plate was coated with 10% pooled human serum in carbonate buffer (15 mM Na_2_CO_3_ and 35 mM NaHCO_3_, pH 9.6, all Sigma) overnight at 4°C. After 3x washes in PBS, the plate was blocked 1% BSA (Sigma) in PBS 1 hr, then washed 3x in PBS + 0.05% Tween-20 (Sigma). From overnight cultures, samples were prepared by filtering supernatant (0.22 μM; VWR) for measures of only released protein, or total culture sonicated for measures of total protein, then each incubated on the plate 1.5 hr. After 3 washes in 0.05% Tween-20, rabbit anti-J8 serum was added 1:100 in PBS 1% BSA and incubated 1 hr. After 3x washes in PBS + 0.05% Tween-20, anti-rabbit-HRP (Sigma) was added 1:200 in PBS 1% BSA and incubated 1 hr. After 5x washes in 0.05% Tween-20, OptEIA TMB substrate (BD) was added and developed following the manufacturer’s protocol. Absorbance was measured on a Victor plate reader (PerkinElmer), with background subtracted from no-sample negative controls. Values were normalized relative to wild-type, non-mutant GAS 5448.

### Gene expression analysis (RT-PCR)

Total RNA was prepared from overnight GAS cultures in THY media and skin lesions collected from mice infected with ΔM1, M1_WT_, M1_SL_, and M1_CS_ for 24 hr. Samples were pelleted and resuspended in equal volume of TRI reagent (ZymoResearch) and lysed by bead beating using 0.1 μm beads. RNA was isolated using DirectZol RNA miniprep kit (ZymoResearch) and RNA Clean & Concentrator kit (ZymoResearch). qRT-PCR was performed using LunaScript RT system (NEB) with oligonucleotides for GyrA forward 5’-GAACGCCAAAGCCAAGCTAT-3’ and reverse 5’-TTGAATCTTATCACGTTCCAAAC-3’, emm1 forward 5’-TCTTGCAGCAAACAATCCCG-3’ and reverse 5’ACGCTGGTCTTCTAAGGCTT-3’. Relative levels of RNA were calculated in M1_WT_, M1_SL_, M1_CS_, and ΔM1 using ΔΔCT method compared to wild-type 5448 GAS for overnight cultures. Relative levels of RNA were calculated in M1_SL_, M1_CS_, and ΔM1 using ΔΔCT method compared to M1_WT_ for *in vivo* samples.

### Cell culture

The human monocyte cell line THP-1 (ATCC) was routinely cultured in Roswell Park Memorial Institute (RPMI) medium supplemented with 10% heat-inactivated fetal bovine serum (FBS) and penicillin/streptomycin. For each experiment, cells were terminally differentiated to macrophages with 200 nM phorbol 12-myristate 13-acetate (PMA; Sigma) for 48 hours, then media replaced with phenol-red free RPMI supplemented with 5% FBS, as previously [77]. 0.4 µm membrane supports (Stem Cell Technologies) were added to each well, submerged in the tissue culture media. To these, bacteria grown overnight in RPMI supplemented with 5% THY were added at MOI 10. After 4 hours of incubation, THP-1 cell supernatants were collected and analyzed for death by Cytox assay (Promega) and for mature IL-1β by IL-1R-lux reporter cells, as previously [78].

### Animal methods

GAS cultures were grown statically overnight at 37°C in THY, washed 2x in PBS, and 10^8^ colony-forming units (CFU) in 100 μL of PBS injected intradermally into seven-week-old C57BL/6 male or female mice (Jackson Laboratories) as previously [39]. Each injection site is prepared by shaving and exfoliated for visualization, and ethanol swabbed immediately before inoculation. Mice were housed in specific pathogen-free conditions with a 14-hour light/10-hour dark cycle in standard ambient environment (~20 °C and ~50% humidity) in ABSL-2 conditions. At indicated times, mice were euthanized with Avertin (2,2,2-tribromoethanol; Sigma-Aldrich) and exsanguinated prior to flow cytometry, or euthanized by asphyxiation prior to excision of lesions. Tissue was homogenized by bead beater for CFU enumeration by dilution plating on THY agar plates and for measurement of cytokines using the V-PLEX Proinflammatory Panel Mouse Kit (Meso Scale Diagnostics) quantified by Meso QuickPlex SQ120 in the Emory Multiplexed Immunoassay Core. For histology, skin lesions were fixed in 4% PFA, paraffinized, sectioned, stained by hematoxylin and eosin (H&E), and imaged by the Cancer Tissue and Pathology Shared Resource of Winship Cancer Institute of Emory University.

### Flow cytometry

Intravital vascular (IV) staining in mice was performed prior to euthanasia. To distinguish immune cells resident in skin from those in circulation, 1.5 µg fluorophore-conjugated anti-CD45 antibody in 200 µl PBS was intravenously injected into the tail vein of mice and allowed to circulate for at least 15 minutes; post-injection, mice were euthanized and skin lesions were digested with hyaluronidase (0.5 mg/ml, Sigma-Aldrich), deoxyribonuclease 1 from bovine pancreas (0.2 mg/ml, Sigma-Aldrich), collagenase from *Clostridium histolytica* (2 mg/ml, Sigma-Aldrich) at 37°C for 1 hr. Samples were cut into 1–2 mm pieces at the end of the first ten minutes of incubation and mixed thoroughly at regular intervals. The samples are passed through a 100 µm cell strainer and rinsed with Dulbecco’s modified Eagle Medium (DMEM) supplemented with 2% FBS. The samples were spun at 450 x g for 10 minutes and red blood cells were lysed using Ammonium-Chloride-Potassium (ACK) lysing buffer (Thermo Fisher). Cells were spun at 520 x g for 5 minutes and resuspended in FACS buffer (PBS supplemented with 5% FBS) for staining. A total of 100,000 immune cells from the skin were stained per sample. Single-cell suspensions were stained for viability using Zombie NIR Fixable Viability Kits (BioLegend), followed by Fc receptor blocking (BioXCell), surface staining (**Table 2**), and measurements using Cytek Aurora. Data was analyzed using FlowJo software (V10.1.1).

### Statistics and data analysis

Values are expressed as mean ± SEM. Differences between groups were analyzed using a 1-way analysis of variance with Dunnett multiple comparisons analysis unless otherwise indicated. Differences are considered statistically significant at a P value of <0.05 using GraphPad Prism v10 software.

## Supporting information

S1 Fig Growth curve of ΔM1, M1_WT_, M1_SL_, and M1_CS_. Data are representative of three independent experiments and are expressed as the mean±SEM.
(EPS)

S2 Fig *emm1* gene expression of engineered strains *in vivo.* qRT-PCR measurements of *emm* gene from mice skin lesions infected with ΔM1, M1_WT_, M1_SL_, and M1_CS_ for 24 hr. Data are representative of three independent experiments. Statistical analysis was done by one-way ANOVA with Tukey’s multiple comparisons test. ****p ≤ 0.0001, ns, no significance.
(EPS)

S3 Fig M protein release changes expression of neutrophil markers. (A) Counts of CD63 + neutrophils and (B) ARG1 + neutrophils per gram of tissue lesion. Data are expressed as the mean±SEM (n = 6). Statistical analysis was done by one-way ANOVA with Dunnett’s multiple comparisons test. *p ≤ 0.05, ns, no significance.
(EPS)

S4 Fig Full cytokine panel of Fig 6B. Statistical analysis was done by one-way ANOVA with Dunnett’s multiple comparisons test. *p ≤ 0.05, **p ≤ 0.01, ***p ≤ 0.001, ****p < 0.0001, ns, no significance.
(EPS)

S5 Fig Conservation of M protein anchoring between strains. (A) Alignment of cell surface proximal (LPXTG) regions of the coding sequences of different *emm*-types; “MK” sequence of M1 protein highlighted. (B) Surface M protein measured in *emm1*, *emm3, emm4* and *emm6* grown to late-exponential phase by flow cytometry. Addition of SpeB inhibitor, E-64 at 200 μM to WT changes M protein levels on GAS surface in *emm1*, *emm3 and emm4*. No M protein was detected for *emm6*. Data are representative of three independent experiments.
(EPS)

S1 File Raw Data for Figures. (XLSX)

S2 Fig Raw images. (PDF)
