# Supplementary figures and images for: Dynamic tethering of M protein drives pathological inflammation during group A Streptococcus infections
Source: PLoS Pathog. 2026 Jul 15;22(7):e1014434. doi: 10.1371/journal.ppat.1014434 (PMC13384396; doi:10.1371/journal.ppat.1014434)

Raw image of Figure 1D

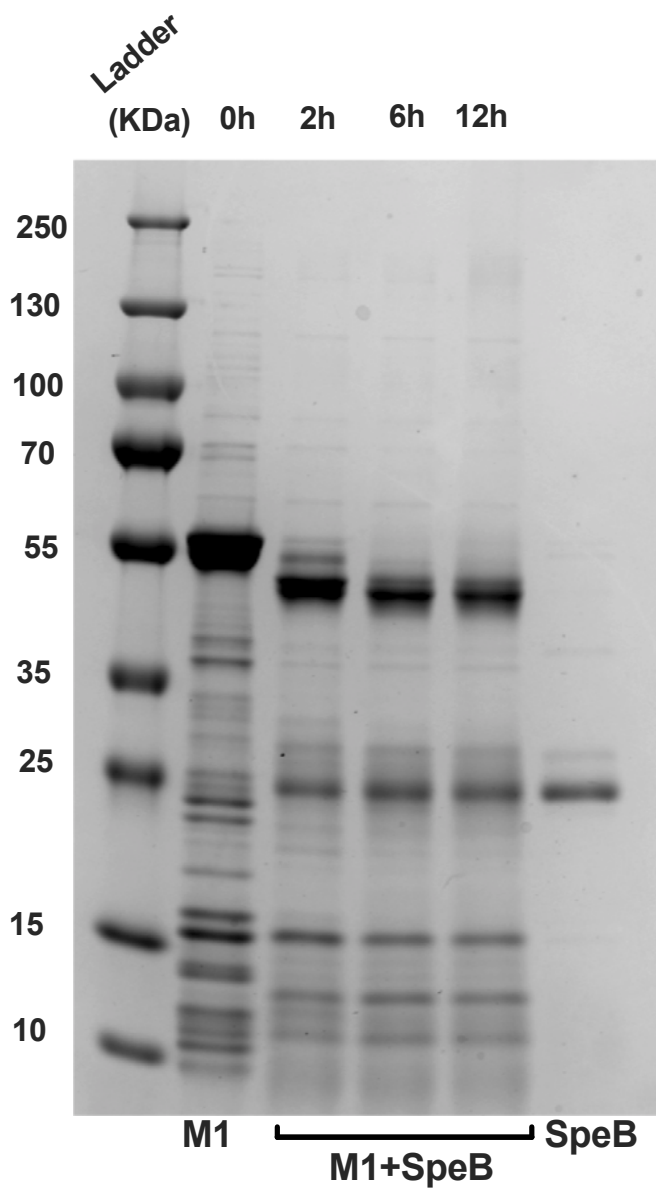

Raw image of Figure 2B

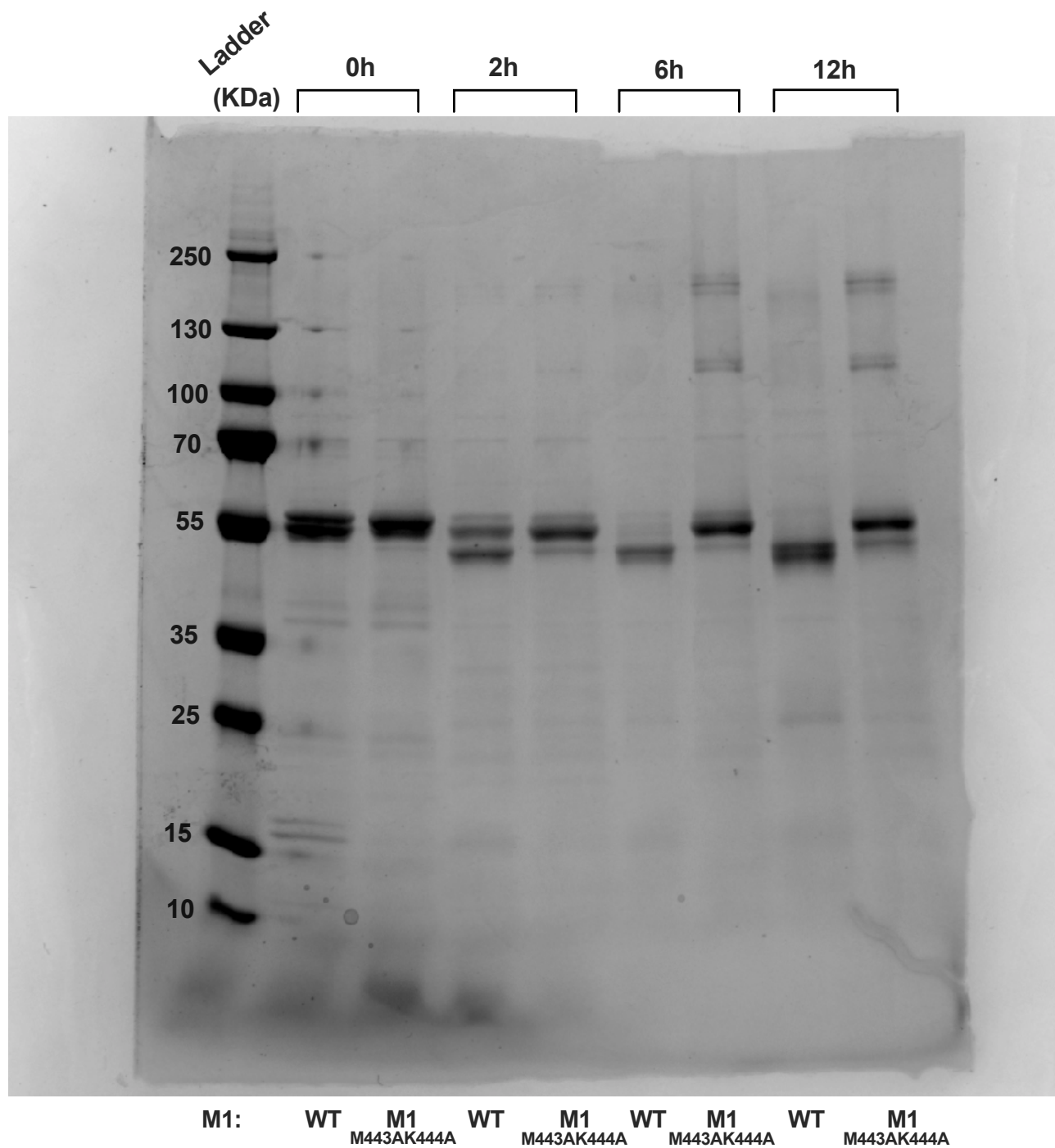

Supplement: S2 Fig — (PDF) [file ppat.1014434.s007.pdf]
